# Understanding the genomic heterogeneity of North African Imazighen: from broad to microgeographical perspectives

**DOI:** 10.1038/s41598-024-60568-8

**Published:** 2024-05-01

**Authors:** Laura Vilà-Valls, Amine Abdeli, Marcel Lucas-Sánchez, Asmahan Bekada, Francesc Calafell, Traki Benhassine, David Comas

**Affiliations:** 1 Departament de Medicina i Ciències de la Vida, Institut de Biologia Evolutiva (CSIC-UPF), Universitat Pompeu Fabra, Barcelona, Spain; 2 Laboratoire de Biologie Cellulaire et Moléculaire, Faculté Des Sciences Biologiques, Université des Sciences et de la Technologie Houari Boumediene, Alger, Algeria; 3 Département de Biotechnologie, Faculté des Sciences de la Nature et de la Vie, Université Oran 1 (Ahmad Ben Bella), Oran, Algeria

**Keywords:** Genetic variation, Biological anthropology

## Abstract

The strategic location of North Africa has led to cultural and demographic shifts, shaping its genetic structure. Historical migrations brought different genetic components that are evident in present-day North African genomes, along with autochthonous components. The Imazighen (plural of Amazigh) are believed to be the descendants of autochthonous North Africans and speak various Amazigh languages, which belong to the Afro-Asiatic language family. However, the arrival of different human groups, especially during the Arab conquest, caused cultural and linguistic changes in local populations, increasing their heterogeneity. We aim to characterize the genetic structure of the region, using the largest Amazigh dataset to date and other reference samples. Our findings indicate microgeographical genetic heterogeneity among Amazigh populations, modeled by various admixture waves and different effective population sizes. A first admixture wave is detected group-wide around the twelfth century, whereas a second wave appears in some Amazigh groups around the nineteenth century. These events involved populations with higher genetic ancestry from south of the Sahara compared to the current North Africans. A plausible explanation would be the historical trans-Saharan slave trade, which lasted from the Roman times to the nineteenth century. Furthermore, our investigation shows that assortative mating in North Africa has been rare.

## Introduction

Over the course of history, North Africa has experienced a series of influential cultural and demographic events due to its strategic position located at the crossroads of three continental regions (Europe, Middle East, and the rest of the African continent), resulting in a complex and varied genetic structure in current populations. These migrations introduced genetic components from the neighboring regions, which are now detected in the genomes of present-day North Africans^1–9^.

Ancient artifacts provide evidence of early hominin presence in North Africa dating back to ~ 2.4 Mya^10^, while the earliest human bones to be directly dated appeared 300 Kya^11^. Although numerous fossils have been discovered in North Africa, Taforalt in Morocco (15,100–13,900 cal bp) is the oldest site for which genetic data is available, with human remains from the Iberomaurusian period in the Epipalaeolithic^7^. Additional Early, Middle and Late Neolithic genomes have also been retrieved from different sites in Morocco^6,8^.

The process of Neolithization in North Africa was introduced by more than one migration wave. In the Early Neolithic, a first migration of European Neolithic farmers introduced farming and cultural knowledge^6,8,12–14^. Genetic analyses, however, revealed that gene flow was unidirectional from autochthonous populations to newcomers^8^. Unlike this first migration, during the Middle Neolithic people migrated from the Levant^8,15,16^ admixed with local populations from North Africa, and introduced a new ancestry, resulting in a heterogeneous landscape by the Late Neolithic. Nevertheless, low levels of genetic diversity and small effective population sizes, likely induced by periods of isolation, may have led to the Maghrebi genetic component that is present today^8^.

The Imazighen (singular Amazigh, also known by the misnomer of Berbers^17,18^), are likely to be the descendants of the autochthonous inhabitants of North Africa, who inhabited the region prior to the demographic movements beginning in the Iron Age ~ 3000 years ago^18,19^. At that time, languages of Afro-Asiatic language family may have already been spoken in the region, with little influence from non-African immigrants^19^. However, significant changes occurred as diverse human groups gradually arrived in the region. The series of historical arrivals began with the influx of Mediterranean-based populations, such as the Phoenicians (~ 900 BCE), Ionians (~ 700 BCE) and Romans (146 BCE)^19^. However, the Arab conquest, beginning in the seventh century, had a profound and lasting cultural impact by introducing the Arabic language and Islam, which plays an important role in the cultural landscape of the region^19^. Despite the fact that Arabization implied a cultural and linguistic assimilation of many autochthonous populations^19^, a wide variety of Amazigh languages that characterize the different groups are still preserved in the region^20^. Besides Arabization, later population contacts such as the Ottoman and European colonial powers also had an influence in the region^19^.

Previous genetic studies using from classical markers to genome-wide data^1–4,6–8,21–26^ demonstrated that North Africa diverges from the rest of the African continent, and more closely resembles out-of-Africa (OOA) populations. Most inhabitants of North Africa carry a mix of four genetic ancestral components, including autochthonous components such as the Maghrebi component^1^. Initial studies suggested that the Maghrebi component could have been introduced in a back-to-Africa movement, more than 12,000 years ago, although an in situ origin cannot be discarded^1,27–30^. The comparison of contemporary genomes and ancient modern human samples from the region supported the presence of the local ancestral component, present in the Epipalaeolithic samples from Taforalt^7^. However, the impact of the Neolithic in North Africa reduced the proportion of this autochthonous component, although it did not erase it^2,8^. Genetic studies have also demonstrated the genetic impact of the Arabic expansion and the trans-Saharan slave trade in current North Africans^2,3,31^, as well as the genetic heterogeneity of North Africa, with different degrees of correlation between cultural background, geographic location, and genetics^3,5,32^.

However, the lower number of genetic studies performed in North Africa compared to other continental territories plus the bias on the sampling in this vast geographic area might have prevented a clear genetic inference of its population history. The limited number of samples analyzed, in addition to the geographically and culturally distant populations under study might have biased some of the conclusions on its past demography. Most of these studies^1–3,6^ have included a single sample population as a representative of a large geographical area (i.e. current political countries) or cultural characteristic (i.e. language spoken), which might have influenced the geographical, cultural, and genetic heterogeneity described in North African populations. In this sense, there are very few examples in North Africa where genetic diversity has been approached with a microgeographical perspective, including different samples from a reduced geographical area and with a variety of cultures and lifestyles^9,32–34^. In order to overcome some of these limitations and to assess the genetic heterogeneity in the region and especially within the Imazighen, we have analyzed genome-wide data from three Amazigh Chaouïa (also spelled Shawiya)-speaking groups from the Aurès region in northeastern Algeria located in a reduced geographic area (~ 2500 km^2^) and present this microgeographical approach in the broad context of genetic diversity in North Africa.

## Results

### Genetic structure in Amazigh populations from a genome-wide perspective

**Figure 1:**
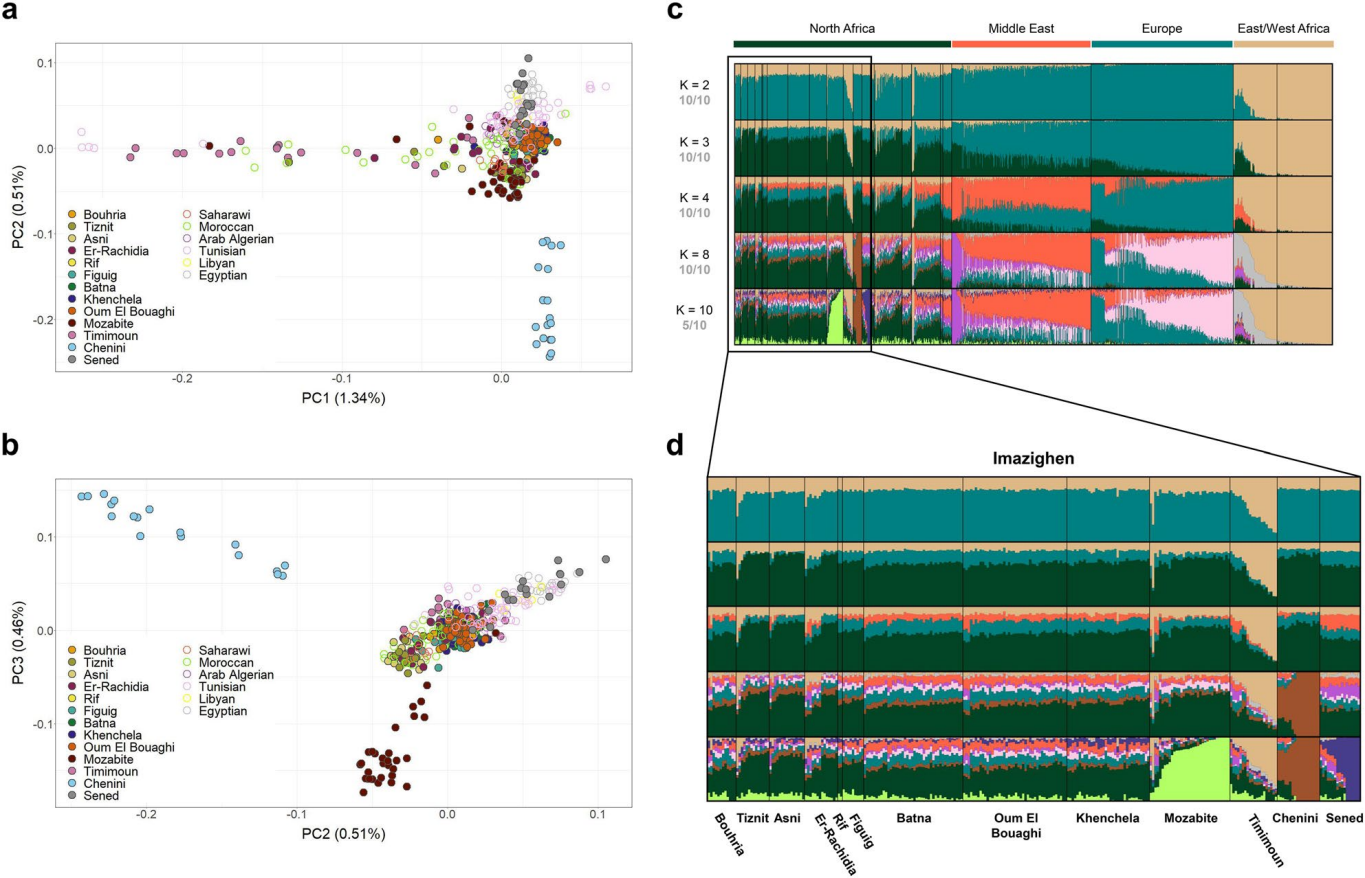
Genetic structure of North African populations using PCA and ADMIXTURE analyses. Panels (**a**) and (**b**) display the PCA results for a subset of North African samples only, plotting PC1 against PC2 and PC2 against PC3, respectively, with Amazigh individuals represented by filled dots. In panel (**c**) is shown the ADMIXTURE analysis for the entire dataset, where K = 4 has the lowest cross-validation error. The plots reflect each K’s major mode. In gray, the number of runs out of 10 classified in the major mode at each value of K. Panel (**d**) shows the ADMIXTURE extended to Amazigh individuals.

We assessed the genetic structure of Amazigh populations, with the largest dataset to date, using genome-wide SNP data from 276 Imazighen (Supplementary Fig. S1) and other North African, sub-Saharan (individuals from the south of the Sahara), Middle Eastern, and European reference individuals, with PCA clustering (Fig. 1a-b and Supplementary Fig. S2) and unsupervised ADMIXTURE analysis (Fig. 1c-d).

A specific component from North Africa appears in the ADMIXTURE analysis (Fig. 1c-d) in varying proportions throughout the region, which is consistent with previous data^1,3^. However, some Amazigh groups exhibit remarkable differences. The Mozabites are separated in the PCA calculated for only the North African samples (Fig. 1b) and present a specific admixture component from K = 10 when considering the whole dataset; Tunisian Imazighen from Sened are also separated from most of the North African individuals in the PCA (Fig. 1b), and show a specific component at K = 10; Tunisian Amazigh individuals from Chenini also cluster separately in the PCA (Fig. 1a-b) and display a distinct component from K = 8 onwards; and Algerian Imazighen from Timimoun present a higher proportion of the sub-Saharan-like component in comparison with the rest of Imazighen and North African reference individuals (Fig. 1c-d), in line with previous studies^3^. In summary, despite the genetic similarities of the Amazigh populations among themselves and among other North African populations in these broad genomic analyses, our results suggest that some Amazigh groups show some degree of genetic differentiation.

To further investigate the genetic structure of the Algerian Amazigh populations, we included North African ancient samples from different archaeological sites spanning the Epipalaeolithic to Late Neolithic^6–8^ and performed a projected-PCA and unsupervised ADMIXTURE analysis. The projected-PCA, shows that ancient individuals fall close to the modern samples from the same region, with the Epipalaeolithic samples being the most separated from the rest (Supplementary Fig. S3a), in agreement with Serra-Vidal et al.^2^. After inferring ancestry components with ADMIXTURE (Supplementary Fig. S3b), we detected from K = 3, also at K = 5 with the lowest cross-validation error, a persistent autochthonous component in the Epipalaeolithic samples from TAF and OUB, and in the Early Neolithic samples from IAM. This component is present in modern North African individuals at intermediate proportions and at lower proportions in the Early Neolithic samples from KTG, the Middle Neolithic samples from SKH and the Late Neolithic samples from KEB, which is consistent with the literature and the disruption of genetic continuity after multiple migration waves during the Neolithic^2,8^.

### Patterns of genetic diversity point to variations at a microgeographical level undetected in global ancestry analyses

**Figure 2:**
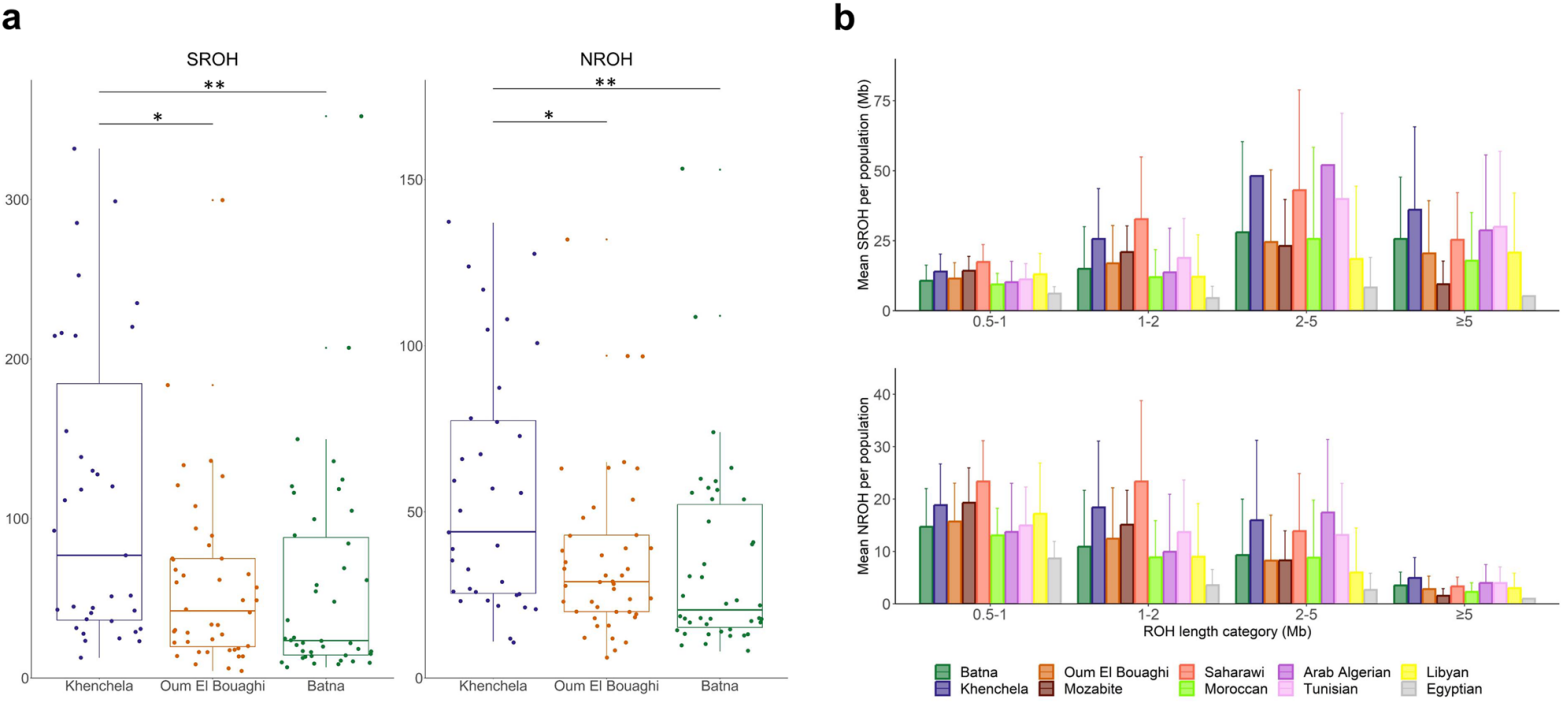
Levels of inbreeding assessed by runs of homozygosity (ROH) > 500 kb. Panel (**a**) compares the total sum (SROH) and the number (NROH) of ROH among the Amazigh populations from the Aurès region. In panel (**b**), the SROH and NROH visualized by different length categories for North African groups.

Although few differences between the three Algerian Chaouïa populations from the Aurès region (i.e. Batna, Khenchela, and Oum El Bouaghi) can be found in our PCA or ADMIXTURE analyses, a closer examination of their levels of inbreeding, defined as the “mating of individuals or organisms that are closely related through common ancestry”^35^, shows significant variations at a microgeographical level (Fig. 2). The Khenchela group has a significantly greater sum (SROH) and number (NROH) of runs of homozygosity > 500 kb (Fig. 2a), after accounting for multiple testing (Supplementary Table S1). When comparing different ROH length categories (Fig. 2b), the Khenchela population not only shows the highest mean SROH in the last category (ROH > 5 Mb), but it also presents a higher NROH of different lengths than the other Chaouïa groups, pointing to historical and current levels of inbreeding. Nonetheless, the differences were only found to be significant in the shorter length categories between Khenchela and Batna (Supplementary Table S2).

Since haplotype-based methods offer a robust fine-scale characterization at a microgeographical level, ChromoPainter and fineSTRUCTURE were used to describe the Algerian genetic substructure in depth and detect fine-scale patterns. FineSTRUCTURE allowed us to group samples in clusters, based on genetic similarity after haplotype inference (Supplementary Figs. S4 and S5, Supplementary Table S3). North African samples cluster in the same branch as European and Middle Eastern individuals, forming a specific branch (Supplementary Fig. S4). After calculating a PCA on the ChromoPainter coancestry matrix, we can see that clustering samples based on genetic similarity from fineSTRUCTURE dendrograms (Supplementary Fig. S5a) is more accurate than the clustering by population label (Supplementary Fig. S5b), especially for North African samples, as some populations present different levels of genetic components, e.g. the sub-Saharan-like component in the Mozabites (Fig. 1c-d), causing them to be separated in the dendrogram despite belonging to the same population.

Algerian Chaouïa samples were mostly distributed in four genetic clusters: *Batna* with only samples from Batna, *OumElBouaghi* with mostly samples from Oum El Bouaghi, *Khenchela* with all samples from Khenchela except one from Oum El Bouaghi, and *OumElBouaghi_Batna* with eight samples from Oum El Bouaghi and four from Batna. The Mozabites formed a unique cluster that we labelled *Mozabite*, although four samples clustered apart. The Algerian Arabs from Algiers were mostly distributed between the clusters *Naf_Mix1* and *Naf_Mix2* together with other non-Amazigh North African individuals (Supplementary Table S3). The above-mentioned clusters were the only ones used in subsequent analyses to address small-scale differences between Algerian populations.

By examining the ancestry profiles per individual obtained with the non-negative-least-squares (NNLS) methods and considering three ancestry-like components (sub-Saharan, European, and Middle Eastern), we identified similarities within the genetic clusters from the Aurès region (Supplementary Fig. S6a,b and Supplementary Table S4), corroborating the global ancestry estimate with ADMIXTURE and the PCA clustering. However, the *Khenchela* cluster has a slightly higher proportion of the Middle Eastern-like component (68.28% ± 2.02) compared to *Batna* (64.82% ± 2.00, *p* value = 3.0 × 10^–7^), *OumElBouaghi* (65.77% ± 2.19, *p* value = 5.4 × 10^–5^) and *OumElBouaghi_Batna* (65.29% ± 2.38, *p* value = 0.004). *OumElBouaghi_Batna* exhibited a larger sub-Saharan-like component (14.88% ± 1.42) compared to the other clusters from the Aurès (*Batna* = 12.68% ± 1.05, *p* value = 1.1 × 10^–4^; *Khenchela* = 12.81% ± 1.44, *p* value = 4.4 × 10^–4^; and *OumElBouaghi* = 12.49% ± 0.95, *p* value = 2.0 × 10^–5^). Notably, the *Mozabite* cluster presents a larger sub-Saharan-like component (17.61% ± 2.39) compared to the other Amazigh genetic clusters under study, but it does not differ from those of the non-Amazigh North African cluster *Naf_Mix2* (17.55% ± 5.98, *p* value = 0.270) (Supplementary Fig. S6a,b and Supplementary Table S4). Additionally, we employed SOURCEFIND, another fine-scale method for admixture modelling, to ensure the robustness of our results. Our findings using this method were consistent with those obtained with the NNLS method (Supplementary Fig. S6c).

**Figure 3:**
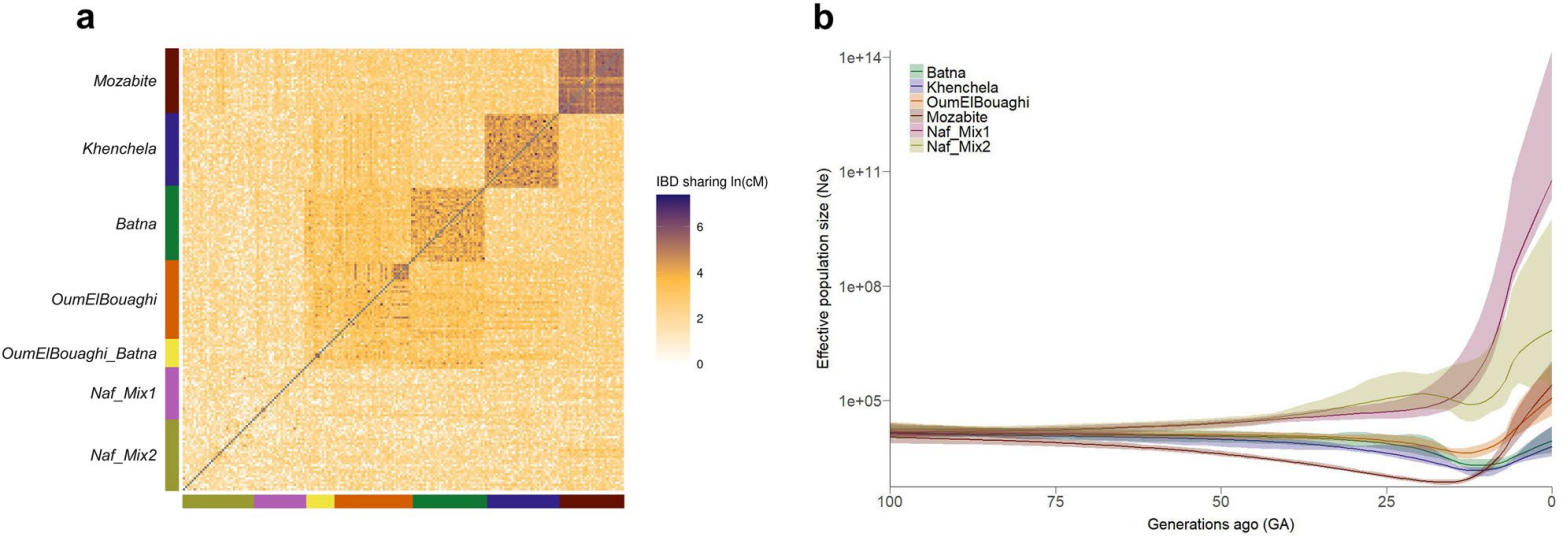
Patterns of genetic diversity inferred from identity-by-descent segments (IBDs). (**a**) Total pairwise IBD-sharing between individuals in natural logarithm, classified in genetic clusters defined with fineSTRUCTURE. (**b**) Effective population size (Ne) through the last 100 generations with 95% confidence intervals, inferred with IBDNe from the IBD segments computed with RefineIBD.

On the other hand, haplotype estimation allowed us to infer the genetic diversity of the clusters of interest, based on identity-by-descent segments (IBDs). The total pairwise sharing of IBD segments (> 2 cM) averaged per population, identified with RefineIBD^36^, indicates that *Khenchela* genetic cluster shares significantly more IBDs within group (92.54 ± 101.14) than the rest of Chaouïa groups: *Batna* (62.75 ± 51.98), *OumElBouaghi* (35.91 ± 40.92) and *OumElBouaghi_Batna* (22.98 ± 42.83) (Fig. 3a, Supplementary Tables S5 and S6), resulting in less genetic diversity in this group. This claim is further supported by the observation of a significantly smaller effective population size (Ne) over the last 100 generations (Fig. 3b and Supplementary Table S7) inferred with IBDNe^37^. The harmonic mean Ne (HMNe) for *Khenchela* is calculated to be 5301, while for the other groups values are larger: *Batna* (HMNe = 7260) and *OumElBouaghi* (HMNe = 10,878) (Supplementary Fig. S7). These results support the observed levels of inbreeding with the runs of homozygosity, for which Khenchela individuals exhibit higher number and total sum of ROHs.

Individuals from Oum El Bouaghi have the lowest IBD sharing within group (Fig. 3a) and the highest Ne among the three Aurès populations (Fig. 3b and Supplementary Fig. S7), but in addition, the average total sum of IBDs shared with Batna (24.54 ± 12.49) and Khenchela (18.08 ± 10.56) is significantly higher than between these two (11.51 ± 6.26) (Supplementary Tables S5 and S6), suggesting that the migration rate between Batna and Khenchela has been smaller than that between either population and Oum El Bouaghi.

In addition, we have detected that the *Mozabite* cluster shows the highest IBD sharing within group (Fig. 3a, Supplementary Tables S5 and S6), and the smallest Ne through time, although the tendency is reversed after an increase in population size from 15 generations ago (ga) (Fig. 3b and Supplementary Fig. S7). These inferences are supported by low levels of recent inbreeding, as measured by the number of long ROHs (Fig. 2b). The reference genetic clusters of non-Amazigh North African populations *Naf_Mix1* and *Naf_Mix2* (Supplementary Table S3) show the lowest IBD sharing (Fig. 3a, and Supplementary Tables S5 and S6) and the highest Ne over time (Fig. 3b and Supplementary Fig. S7).

### Assessing the complex interplay of admixture and genetic diversity in Amazigh populations

Admixture can bias metrics such as IBD segments and ROH, often used to measure genetic diversity or infer population history. To test this, we inferred first the admixture timing and the source populations involved in the admixture with GLOBETROTTER^38^, for target genetic clusters defined with fineSTRUCTURE (Supplementary Table S3)^39^.

We found evidence of multiple waves of admixture in Amazigh groups, consistent with previous studies^3^. Using GLOBETROTTER, we detected multiple dates of admixture in three Amazigh genetic clusters and only one date in the other groups tested, including two Amazigh and two non-Amazigh genetic clusters. Due to the limitations of the method, to address the multiple dates inference, we could only infer two admixture dates that should be interpreted with caution since it is difficult to differentiate between continuous gene flow and independent admixture events.

**Figure 4:**
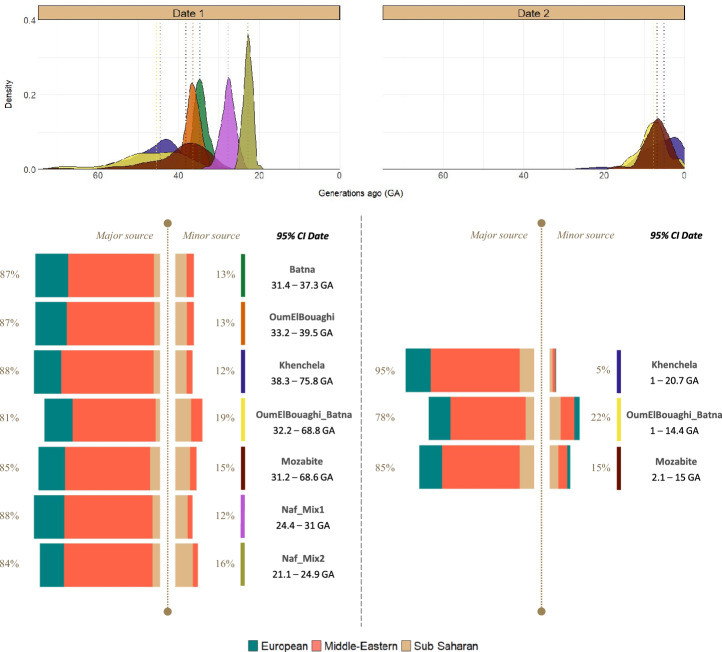
Admixture events inferred in North African genetic clusters. This figure shows the admixture events inferred in the target North African genetic clusters, including the 95% CI of the dates inferred, the composition of the source populations participating in the admixtures and their contributions.

The oldest admixture, common to all the groups, is placed around 35.7 ga (1130 CE); however, the admixture timing differs considerably between populations, with the clusters with two admixture events inferred showing a wider admixture time range (Fig. 4). This admixture event involved a major source population with a major component (71%) from the Middle East, and European-like and sub-Saharan-like components, with average proportions of 23.3% and 5.8% respectively. The minor source population participating in this admixture event contains on average 67.4% of the sub-Saharan-like component and 32.6% of the Middle Eastern-like component. The most recent admixture, only inferred for *Khenchela*, *OumElBouaghi_Batna* and *Mozabite* genetic clusters, is placed around 6.6 ga (1858 CE) and involved a major source population with an average 69.4% of the Middle Eastern-like component, 20.1% of the European-like component and 10.5% of the sub-Saharan-like component. The minor sources inferred for this event present a higher proportion of the sub-Saharan-like component (41.8%), while the average proportions of the Middle Eastern and European-like components are 45.7% and 12.6% respectively (Fig. 4). The similar genetic composition of the sources between the two inferred events, formed by the same surrogate populations with slightly different percentages, is compatible both with multiple waves and with continuous admixture during the inferred time ranges (Supplementary Fig. 8).

The *Khenchela* and *Mozabite* genetic clusters, for which we detected multiple dates of admixture, match the Amazigh populations with more homozygous positions in the genome and higher IBD sharing (Supplementary Tables S1 and S5). To address whether the latest admixture is responsible for the low levels of genetic diversity in these groups, we inferred the hypothetical ancestry of each IBD segment, using local ancestry tracts^40^, to calculate the effective population size per ancestry (asIBDNe)^36^. If the elevated number of IBD segments in these groups was associated to a specific ancestry, we could contrast this result with the detected ancestry proportions of each individual and the inferred components of the source populations participating in the admixture.

Even though we also inferred two admixture dates for the genetic cluster *OumElBouaghi_Batna*, it was not possible to estimate the asIBDNe because of the low number of IBD segments detected, probably due to a smaller sample size (N = 12).

The asIBDNe over time per ancestry shows no particular feature in the *Khenchela* and *Mozabite* clusters (Supplementary Fig. S9a). The three ancestries tested display a similar tendency, resembling the total Ne inferred (Fig. 3b), suggesting that the genetic homogeneity within *Khenchela* and *Mozabite* clusters is not associated to any specific ancestry.

To confirm this, we calculated the age of the ancestry-specific IBD segments to find out whether they were prior or posterior to the admixture. More precisely, we used the equation s19 from Al-Asadi et al.^41^: *E* = *75 x (2/L)*; following the steps in Alva et al.^42^, to calculate the expected length of an IBD segment in centiMorgans (cM) at the beginning of the most recent admixture. This way, we classified IBDs in two groups: shorter or longer than this calculated length, equivalent to IBDs inherited before or after the admixture start. The threshold length for *Khenchela* is 7.25 cM and for *Mozabite* is 10 cM.

We observed no increase in number of IBDs in *Khenchela* and *Mozabite* clusters following the admixture event for any of the three ancestries tested (sub-Saharan, European, and Middle Eastern) (Supplementary Fig. S9b). Indeed, the number of IBDs per ancestry is correlated with the proportion of the respective ancestry in the group (Supplementary Fig. S6).

Since it was not possible to differentiate between ancestries with both asIBDNe and the number of IBDs inherited after the admixture, we cannot demonstrate that the low levels of genetic diversity in the Mozabite and Khenchela groups are linked to the most recent admixture detected with GLOBETROTTER, as some degree of difference would be expected due to the different proportions of ancestry components of the inferred source populations participating in the admixture.

### Inference of nonrandom mating in North Africa by analyzing parental proportions of sub-Saharan ancestry

**Figure 5:**
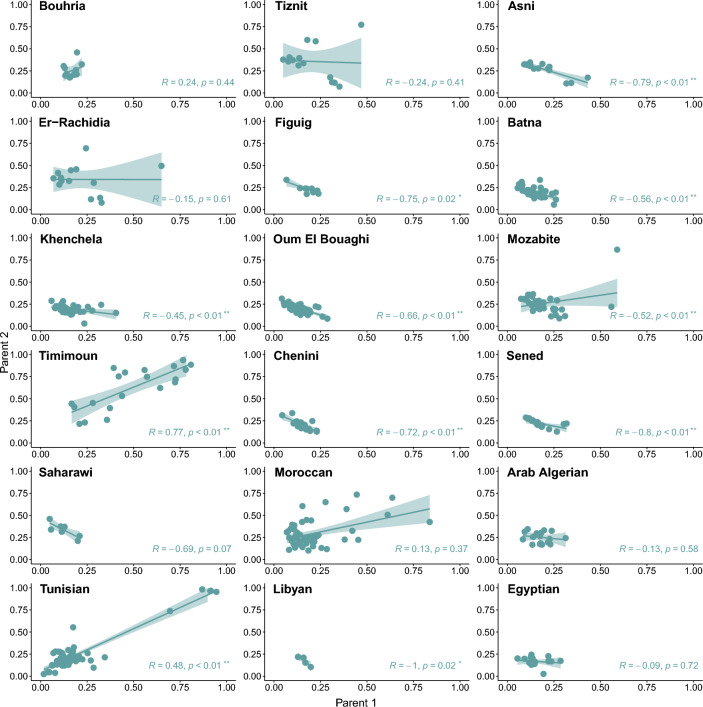
Nonrandom mating inference in North Africa for the sub-Saharan-like ancestry. Nonrandom mating estimates seen as the correlation of the parental ancestries. The linear regression line, the 95% confidence interval, the Spearman correlation coefficient (R) and the p-value are represented in each plot, where * indicate *p* values between 0.01 and 0.05 and ** *p* values < 0.01.

To test for nonrandom mating in both Amazigh (N = 12) and reference North African (N = 6) populations, we analyzed if pairs of mating individuals correlate in sub-Saharan-like genomic ancestry. Note that this analysis was not performed for the Rif population due to the small number of samples (N = 2). We focused on the sub-Saharan ancestry because it is easier to track in local ancestry inferences than other ancestries such as European or Middle Eastern-like, which diverged more recently. For this purpose, after first inferring local ancestry with RFMix^40^, we estimated the parental ancestry proportions that resulted in the ancestry proportions of the current generation with ANCESTOR^43^. We detected nonrandom mating in 11 out of 18 groups, seen as significantly positive or negative Spearman’s correlation of the inferred parental ancestries. Nine populations show a significantly negative Spearman’s correlation coefficient, which allows to reject the hypothesis of assortative mating in North Africa. In fact, this implies a phenomenon known as disassortative mating or negative assortative mating, where mating occurs between individuals who are more phenotypically dissimilar than would be expected by chance^44^. However, the two groups that display a significant positive correlation, Tunisian Arab and Imazighen from Timimoun, coincide with the two populations with more individuals with higher sub-Saharan-like ancestry. This finding could point to insufficient statistical power due to small sample sizes but can also be attributed to the narrow range of ancestry proportions within-group, which are generally low in the region (Fig. 5).

## Discussion

Our study provides a broad understanding of the genetic complexity of North Africa through an extensive analysis of 13 Amazigh groups, which represents the largest dataset to date including inhabitants of the Tamazgha (the Amazigh territory). Our findings have yielded several important conclusions: the presence of distinct genetic components and varying levels of isolation among Amazigh groups, pointing to differences in their demographic histories, multiple admixture events in some Amazigh groups and no evidence of assortative mating in most North African populations under study.

However, it is essential to acknowledge certain limitations in our study. Due to differences in the DNA technology platform used, the number of overlapping Single Nucleotide Polymorphisms (SNPs) was insufficient to perform haplotype-based analyses with the *Complete dataset* (see Methods). Another challenge lies in inferring the Maghrebi component, which is specific to North Africa, as the current population of the region is a combination of several ancestry components, leading to the absence of a good proxy for the Maghrebi component. Consequently, we had to infer local ancestry without accounting for this component, with the added challenge of differentiating the rest of the components, as some diverged in very recent times. An alternative to this challenge would be the incorporation of ancient genome data in haplotype-based methods. However, the number of SNPs shared between the ancient samples and the genotyping array used is not enough for these methodologies. Complete present-day genomes with enough coverage would be needed to have sufficient SNPs to compare with ancient genomes, and, unfortunately, few genomes are available in the region^2,45–47^.

Our findings support the notion that North Africa has a complex demographic history and that its inhabitants are genetically heterogeneous. Confirming the evidence from earlier research, we have detected an autochthonous genetic component, in all North African individuals, in addition to other components from Middle East, sub-Saharan Africa, and Europe^1,3^, in agreement with the mitochondrial haplogroups identified in the Aurès^33^. After incorporating the ancient North African samples, we can confirm the presence in modern samples from the region, including the Algerian Imazighen, of an ancestral component that existed 15,000 years ago, despite the impact of Neolithization, previously observed by Serra-Vidal et al.^2^.

Despite the cultural differentiation between Arab and Amazigh groups in North Africa, we have not identified significant genetic distinctions between these populations, in line with previous genetic studies^3,23,24,48–52^, although other studies that incorporate a functional focus to the analyses, have found significant differences between North African populations (including those from the Aurès, here studied) linked to their cultural background^5,32^. Nevertheless, as also shown in refs. 1 and 3, some Amazigh populations from Tunisia, differ from the others, which can possibly be explained by geographical isolation and little contact with other populations. Moreover, Imazighen from Timimoun have been more in contact with sub-Saharan populations, compared to the other Algerian Amazigh groups studied^3^. Nonetheless, these previous studies included (culturally and geographically) distant populations in the vast and underexplored North African region, and the degree of singularity of some populations might be related to the lack of geographically close samples for comparison. In order to overcome this limitation, we aimed to explore this genetic heterogeneity at a microgeographical level (a geographical area of 2500 km^2^), analyzing some close Algerian Chaouïa-speaking Amazigh populations.

Although there are no apparent distinctions in the proportions of genetic ancestral components between the three Chaouïa-speaking Amazigh groups from Algeria, subtle variations emerge when examining levels of inbreeding and within-group genetic similarity. Previous studies have found that the maternal lineages of the inhabitants of the Aurès region are dominated by West Eurasian lineages with a modest presence of North African (M1/U6) and sub-Saharan (L) lineages^33^. Furthermore, a distinct degree of isolation across the populations under study has been identified, as well as a higher mutational load under a recessive model of dominance in those groups more isolated^32^. In the present study, we detected that Khenchela individuals share more identity-by-descent segments among themselves and exhibit a greater frequency and extent of homozygous regions of varying lengths, suggesting that Imazighen from Khenchela have experienced greater historical and current levels of isolation. These results are also supported by a lower effective population size (Ne) through time, and a higher mutational load under a recessive model, detected in^32^.

We compared these findings with historical population data from each city and its corresponding region, referencing official censuses from 1966, 1977, 1987, and 2008, by the *Office National des Statistiques* in Algeria. In 1966, the wilaya (province) of Khenchela had 13.1 inhabitants per square kilometer (h/km^2^), the wilaya of Batna had 30.6 h/km^2^, and the wilaya of Oum El Bouaghi had 34 h/km^2^. Two decades later, in the census of 1987, the population of the wilaya of Batna surpassed that of Oum El Bouaghi, and this pattern persisted in the most recent official census in 2008, with wilayas’ population sizes of 386,683 (39.4 h/km^2^) in Khenchela; 1,119,791 (91.9 h/km^2^) in Batna; and 621,612 (91.6 h/km^2^) in Oum El Bouaghi. Urbanization rates also changed, with Khenchela going from 28.5% in 1977 to 68.08% in 2008, Batna from 34.1 to 61.17%, and Oum El Bouaghi from 25.24 to 73.15%, indicating a faster growth and a more relevant demographic movement to the cities in the wilaya of Oum El Bouaghi^53^.

Despite Khenchela’s demographic growth in the latter half of the twentieth century, it remained as the least populated. Oum El Bouaghi, despite not currently holding the largest population, exhibited the highest genetic diversity. This phenomenon might be attributed to its historical population size, as suggested by the initial official censuses showing a higher number of inhabitants per square kilometer^53^, favored by its accessibility, since it is situated in a less rugged area compared to Khenchela and Batna. These observations suggest that the geography and accessibility of the mountainous area may have determined the isolation levels of each population, with Oum El Bouaghi being the most accessible and having lower levels of genetic similarity. Additionally, considering Batna’s historical status as the capital of the Aurès, it likely had more connections than Khenchela^54^.

On the other hand, the Mozabites showed the highest IBD sharing within group, and higher levels of historical inbreeding, although these have decreased considerably over time, as seen with the runs of homozygosity analyses. This finding is corroborated by a small Ne that reversed its tendency and began to increase dramatically 15 generations ago. Mozabites inhabit the M’zab Valley, which is a hot arid area located in the northern part of the Sahara Desert in Algeria. Its geographic, climatic and cultural conditions could have limited access to the region, increasing the levels of isolation. However, The M'zab Valley has experienced a much accelerated urban and demographic growth during the nineteenth century, which could explain the recent increase in population size^55,56^.

The inference of admixture events revealed that North Africa hosted the mixing of different populations during a long period of time, in accordance with historical demographic movements and previous research^3,9,31^. Our findings suggest as well different gene flow processes in Amazigh and non-Amazigh populations. The oldest admixture event, inferred in all groups studied, was dated to ~ 1100 CE, with very wide confidence intervals in some populations, starting as early as the second century CE. Even so, a second, more recent admixture event dated to ~ 1800, was inferred in some Amazigh groups for which we detected multiple dates. The genetic make-up of the source populations participating in the admixture events inferred suggests that both events could be associated with the trans-Saharan slave trade, compatible with a continuous admixture. The latter occurred mostly after the Arab world established commercial interactions with sub-Saharan Africa, and had its peak between the eighth and sixteenth centuries CE. However, it is most likely that the trans-Saharan slave trade began around 1000 BCE, becoming an important factor in the Carthaginian economy^57^. It was not until the mid-nineteenth century that slave trade and slavery were banned in North Africa, being legally abolished in Algeria in 1848, following French colonization^58^. Therefore, our date estimates, placed around 1130 CE the oldest and 1858 CE the most recent, and involving minor source populations with a large sub-Saharan component, could be linked to the contacts between inhabitants of North Africa and enslaved sub-Saharan individuals. The former coincides with the peak of the Arab slave trade, and the latter happened a decade after abolition in Algeria^57,58^. Although our findings are consistent with historical records, additional information is required to corroborate the association between the inferred admixture pulses and the migrations that occurred in North Africa, as well as to distinguish whether it was a continuous flow or multiple admixture events over the estimated time.

Despite the fact that we detected multiple admixture dates for the Khenchela and Mozabite groups, they exhibit the lowest levels of genetic diversity. However, we were unable to demonstrate that the latter are related to the most recent inferred admixture event, unique to these populations. Although the participating source populations may have undergone isolation prior to the admixture, isolation is not directly responsible for the current genetic similarity within Khenchela and Mozabite populations.

While assortative mating based on parental ancestries has been identified in admixed populations with sub-Saharan and European ancestries^59^, our analysis reveals that this pattern is infrequently observed in North African populations. Specifically, assortative mating was only identified in two populations, in contrast with disassortative mating observed in nine. Furthermore, assortative mating was detected in populations with a wide range of sub-Saharan-like ancestry proportions. This suggests that non-random mating can vary on a small geographical scale, potentially reflecting cultural practices. However, the absence of positive correlations between parental ancestries in most North African groups could be attributed to small differences in within-group ancestry proportions, and low overall levels of these proportions, as well as insufficient statistical power.

Despite the considerable progress made in clarifying the demographic history and genetic diversity of North Africa, existing gaps can be addressed in order to achieve a more comprehensive and representative picture. The claimed genetic heterogeneity of North African populations has been challenged by the lack of data in this vast region, and future studies with larger sample sizes and whole genome sequences, covering the whole geographical area, cultural aspects, and habitats will contribute significantly to verify earlier findings and to discover previously unknown aspects about their complex demographic history.

## Materials and methods

### Datasets and quality controls

Samples from five Algerian groups were collected from: Amazigh individuals from the cities of Batna (n = 47), Oum El Bouaghi (n = 47) and Khenchela (n = 39)^32,33,60,61^**,** Mozabite Amazigh individuals from Ghardaïa (n = 16) and Arab individuals from Algiers (n = 40)^32^. The cities of Batna, Oum El Bouaghi and Khenchela are the capitals of the wilayas (provinces) of the same name, located in the mountainous region of the Aurès in Northeastern Algeria, characterized by a rugged topography^54^.

Unrelated volunteers, with appropriate informed consent, received information about the objectives of the project, which is centered on the genetic study of the Algerian population. The study complies with the ethical rules of all the institutions involved and has been approved by *the Laboratoire de Biologie Cellulaire et Moléculaire at the Faculté des Sciences Biologies* of *Université de Sciences et Technologie Houari Boumedienne* in Algiers and the CEIm-PSMAR IRB in Barcelona (2019/8900/I). All methods in this study were performed following the standard guidelines and regulations in accordance with the Declaration of Helsinki. Moreover, members of the research team made sure to clarify any doubts the participants might have. Amazigh participants from the Aurès were Chaouïa-speakers born in the allocated city, and whose parents and grandparents were born in the region. The Mozabite and Arab participants were Mozabite and Arabic-speakers, respectively. More information about the study and preliminary data of the current samples were presented in Bekada^62^ and Abdeli^63^.

Samples were genotyped with the Affymetrix Axiom Genome-Wide Human Origins 1 Array, which targets ~ 629,000 SNPs^46^. Genotype calling was performed with the Axiom Analysis Suite 4.0.3 software using default settings for thresholds. 46 Batna, 47 Oum El Bouaghi, 37 Khenchela, 14 Mozabite and 34 Algerian Arab samples passed the genotype calling with an average quality control rate of 99.8%, and were deposited in EGA under accession number EGAS00001007235^32^.

Quality Control filtering was done with PLINK/1.9b^64^ to remove variants with > 5% of missingness, individuals with > 10% of missingness, SNPs failing the Hardy–Weinberg exact test with a *p* value < 10^–5^, third-degree or closer related individuals (PI_HAT > 0.125). A total of 26 samples were removed after the latter filter: Batna (n = 4), Oum El Bouaghi (n = 3), Khenchela (n = 2), Mozabites (n = 1) and Arabs (n = 16).

Two datasets were created for this study to address different questions. For a more complete view of Amazigh genetics, demography, and history, we built a dataset with all the samples available from Imazighen (Supplementary Fig. S1)^2,3,34^, non-Amazigh groups in North Africa, and reference samples^31,46,47,65–71^ (Supplementary Table S8). The QC described above was applied to each individual dataset before merging them. If necessary, duplicated variants were removed and some variants were flipped due to DNA-strand inconsistency. Once all the samples were merged, SNPs with a minor allele frequency < 1% were removed and a linkage disequilibrium (LD) was pruned by using a window size of 200 SNPs, shifting by 25 SNPs, and a maximum pairwise LD threshold (r^2^) of 0.5, resulting in 37,019 variants (*Complete dataset*).

For more microgeographical inference focused on Algerian individuals, we built a dataset with reference samples genotyped with the same array or sequenced for whole genomes^31,46,47,65–71^ (Supplementary Table S9). Before merging the data, QC filters for missingness, Hardy–Weinberg equilibrium and relatedness were applied to the reference dataset, and after the merge and the filter for MAF, 397,760 variants remained (*HO dataset*) and 201,448 variants after pruning (*HO dataset pruned*).

### Population structure

To investigate the population structure of Amazigh populations, we have performed for the *Complete dataset* a Principal Component Analysis (PCA) with SmartPCA from EIGENSOFT v6.0.1^72^ and an unsupervised ADMIXTURE v1.3.0^73^, for 10 independent iterations, with K ancestral components from 2 to 10. We used PONG v1.4.9^74^ in order to combine the different iteration results, obtain the major modes for each K value and plot them.

To further investigate the population structure of the Amazigh populations, we merged the *HO dataset* with North African ancient samples from the Epipalaeolithic sites of Taforalt (TAF) and Ifri Ouberrid (OUB), the Early Neolithic sites of Ifri n’Amr o’Moussa (IAM) and Kaf Taht el-Ghar (KTG), the Middle Neolithic site of Skhirat-Rouazi (SKH) and the Late Neolithic site of Kehf el Baroud (KEB)^6–8^. TAF, IAM and KEB ancient samples were obtained from the Allen Ancient DNA Resource (AADR, v54.1.p1)^75^. We kept the common variants between datasets, a total of 205,694 SNPs in 1,182 individuals. In addition, we removed those variants with > 5% of missingness, a MAF < 5% and in linkage disequilibrium (LD), considering a window size of 200 SNPs, shifting by 25 SNPs, and a maximum pairwise LD threshold (r^2^) of 0.5, resulting in 111,324 SNPs.

We performed a projected Principal Component Analysis (PCA) with SmartPCA from EIGENSOFT v6.0.1^72^ and an unsupervised ADMIXTURE v1.3.0^73^, for 10 independent iterations, with K ancestral components from 2 to 10. We used PONG v1.4.9^74^ to plot the ADMIXTURE results.

### Estimation of admixture timing

To explore admixture events at a microgeographical level, we used first SHAPEIT v2^76^ for haplotype estimation in the *HO dataset* with the HapMap phase III^77^ genetic map and the 1000G dataset^78^ as reference panel. 894 variants were removed due to mismatches.

ChromoPainter^39^ was used to estimate the haplotype-sharing between individuals, reconstructing each recipient sample as a mosaic of haplotypes from donor samples. The first time, we estimated the global mutation probability (M) and the switch rate (n) parameters, in chromosomes 1, 7, 14, and 20, for 15 iterations of the expected-maximization (EM) algorithm, with parameters -in -iM. The parameters obtained were averaged across chromosomes weighting by the number of SNPs per chromosome, to obtain the final switch rate (n) and global mutation probability (M), being 262.34 and 0.000852, respectively.

Sequentially, fineSTRUCTURE v2.1.0^39^ was used to organize the samples into homogenous genetic clusters. It was performed for the chunkcounts sharing coancestry matrix from ChromoPainter, for three different seeds and with the following parameters: 2 million Markov Chain Monte Carlo (MCMC) iterations, 1 million burn-in iterations, and sampling values every 10,000 iterations. FineSTRUCTURE dendrograms were built with the default parameter -m T. Samples were assigned into one genetic cluster after checking the consistency of the dendrograms between different seeds (Supplementary Fig. S4c and Supplementary Table S3). Individuals located far from the branches of their population were assigned to other clusters. In those cases where there are branches that are not dominated by any population, the clusters were named according to their geographical origin followed by the label "Mix". In addition, we computed a PCA on the chunkcounts coancestry matrix (Supplementary Fig. S5)^39^. Then, we ran ChromoPainter a second time indicating which genetic clusters would perform as donor groups (Supplementary Fig. S4 and Supplementary Table S3).

To infer and date admixture events, we used GLOBETROTTER^38^. First, we set *null.ind* to 1 to estimate p-values for admixture evidence. Second, we set the same parameter to 0 to estimate proportions, dates, and sources of admixture. The date estimates were obtained after performing 100 bootstrap iterations. After that, the 95% confidence intervals (CI) were inferred. We considered 25 years per generation to infer dates in years. The haplotype sharing proportions of the different genetic clusters per individual were inferred with the non-negative least-squares (NNLS) method and SOURCEFINDv2^79^.

### Estimation of population effective sizes

To observe differences between populations effective sizes, we computed first the runs of homozygosity (ROHs) for the *HO dataset pruned*, to detect level of past and recent inbreeding in the target populations. ROHs were estimated with PLINK 1.9b^64^, considering those with at least 50 SNPs, a minimum length of 500 kb and a maximum gap between two consecutive SNPs of 100 kb.

To estimate the effective population size (Ne) through generations and explore the IBD sharing between and within genetic clusters, we used RefineIBD^36^, IBDNe^37^ and asIBDNe^80^, considering a minimum length of 2 cM in all the analyses.

First, the *HO dataset* was phased with Beagle 5.1^81^, with the GRCh37^82^ genetic maps. Second, we ran RefineIBD^36^ to infer IBD segments. The IBD sharing was calculated by summing the IBD pairwise lengths between individuals^83^. Third, IBDNe^37^ was run using the IBDs obtained with RefineIBD.

To run asIBDNe we followed the pipeline from Browning et al.^80^, using RFMix v1.5.4^40^ to identify the ancestry of genomic segments, considering three ancestries for the reference samples: sub-Saharan-like, European-like and Middle Eastern-like. We estimated the age of the ancestry-specific IBD segments obtained from this method, following the steps used in Alva et al.^42^, using the equation s19 from Al-Asadi et al.^41^: *E* = *75 x (2/L)*, where *E* is the start of the admixture event or the time to the most recent common ancestor in generations ago (ga), and *L* is the length of the IBD segment in centiMorgans (cM).

### Inference of parental ancestries and nonrandom mating

We used ANCESTOR^43^ to infer the parental ancestries and test for ancestry-assortative mating. For that, ANCESTOR requires phased local ancestry tracts, which were obtained with RFMix v1.5.4^40^ considering two ancestries for the reference samples: sub-Saharan-like and non-sub-Saharan-like. To convert the outputs from RFMix to the required inputs for ANCESTOR, we used the scripts from Korunes et al.^59^. Nonrandom mating was tested for the sub-Saharan like ancestry by correlating the parental ancestries (Parent 1 vs. Parent 2).

Initially, this analysis was performed for the phased data of the *HO dataset*, obtained with Beagle as described above. However, we aimed to test for ancestry-assortative mating in the Amazigh populations previously published in Arauna et al.^3^ and Hernández et al.^34^. Thus, we merged the samples of interest from each dataset, which were genotyped with different arrays, with publicly available whole-genome data^47,78^. First, we applied the previously described QC filters for missingness, Hardy–Weinberg equilibrium, relatedness, and duplicated variants for the reference datasets, before merging them. Once we had a unique reference panel, we merged it with both datasets of interest and filtered for MAF. The final numbers of variants for the Arauna et al. (2017) and Hernández et al. (2020) datasets were 440,565 and 1,469,713, respectively.

Subsequently, both datasets were phased with Beagle 5.1^81^, the local ancestry tracts were obtained with RFMix v1.5.4^40^ and the parental ancestries were inferred with ANCESTOR^43^, following the exact same steps explained above.

### Supplementary Information

Supplementary Figures.

Supplementary Tables.

## Data Availability

Genome-wide array data of Algerian Imazighen and non-Imazighen are available at the European Genome-phenome Archive (EGA), under accession number EGAS00001007235.
