# Supplementary material for: Understanding the genomic heterogeneity of North African Imazighen: from broad to microgeographical perspectives
Source: Sci Rep. 2024 May 1;14:9979. doi: 10.1038/s41598-024-60568-8 (PMC11063056; doi:10.1038/s41598-024-60568-8)
Supplement: Supplementary file 1 — Supplementary Figures. [file 41598_2024_60568_MOESM1_ESM.pdf]

Supplementary Figures

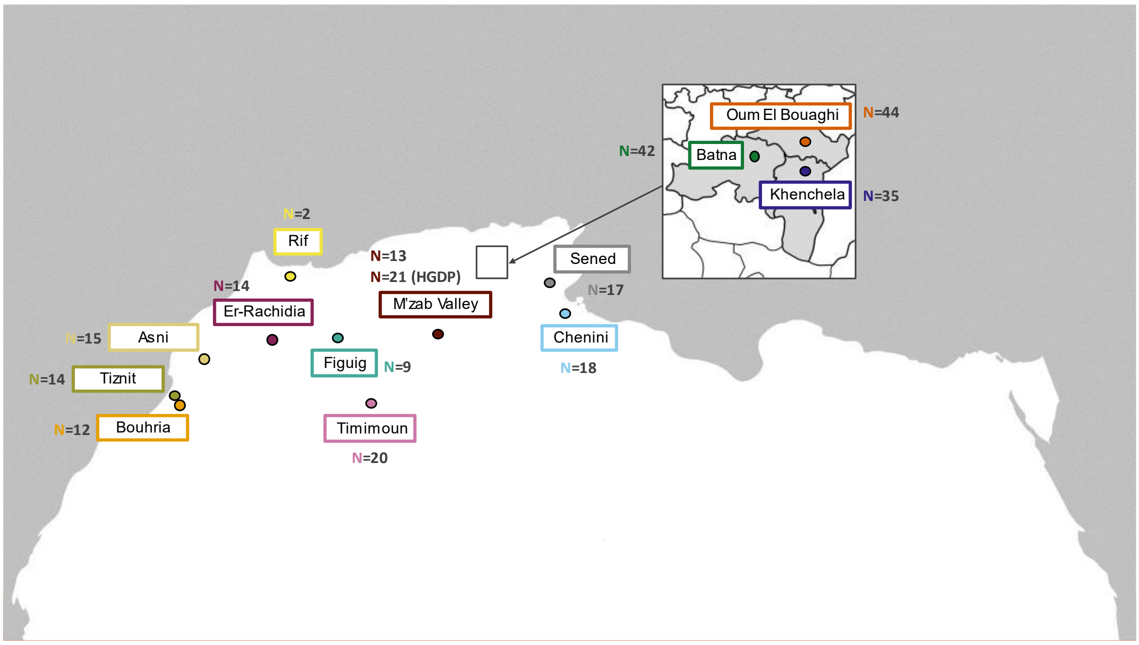

Supplementary Figure S1. Map including all the Amazigh groups from North Africa, their location and sample size after the Quality Control (QC). Bouhria, Asni and Figuig samples are from Hernández et al. (2020), Tiznit, Er-Rachidia, Timimoun, Sened and Chenini samples are from Arauna et al. (2017), Rif samples are from Serra-Vidal et al. (2019), some Mozabite samples are from Patterson et al. (2012) and Mallick et al. (2016) and Batna, Oum El Bouaghi, Khenchela and the other Mozabite samples are from Lucas-Sánchez et al. (2024).

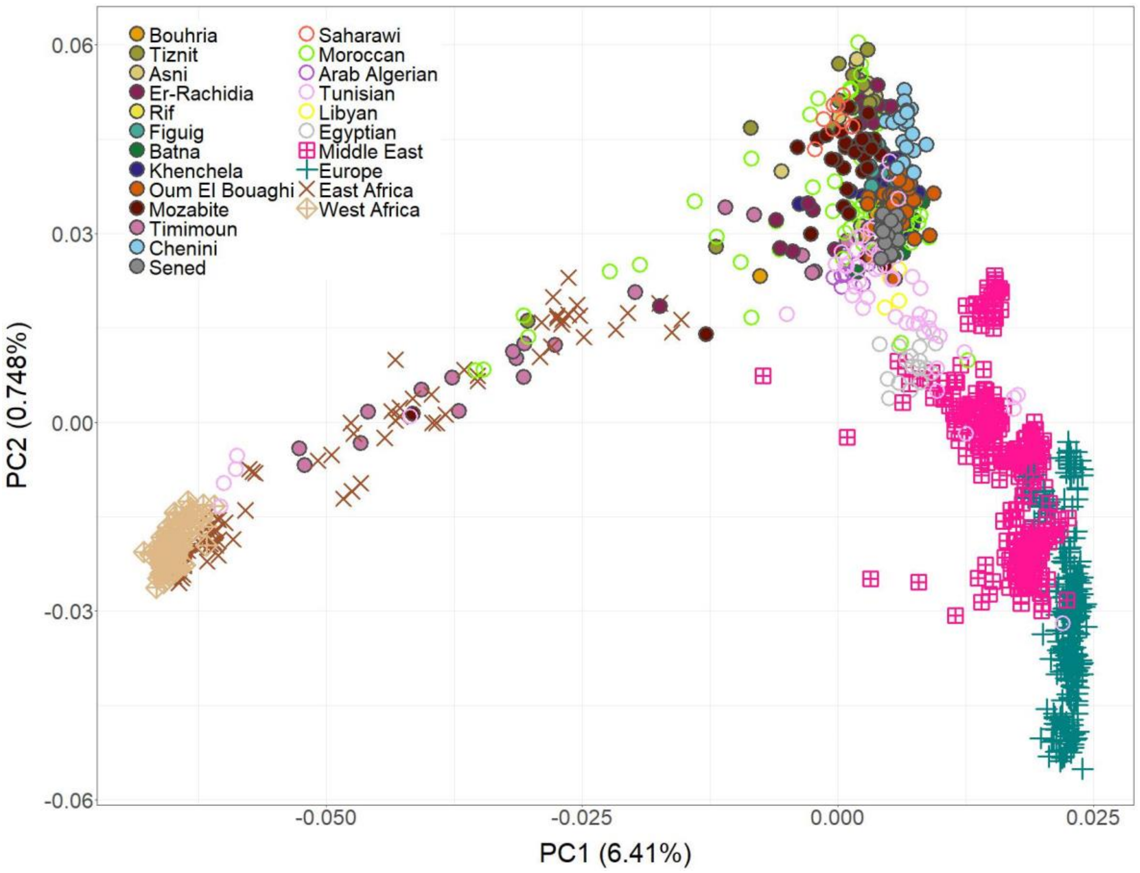

Supplementary Figure S2. PCA results for the *Complete dataset* (see Methods), plotting PC1 against PC2, with Amazigh individuals represented by filled dots.

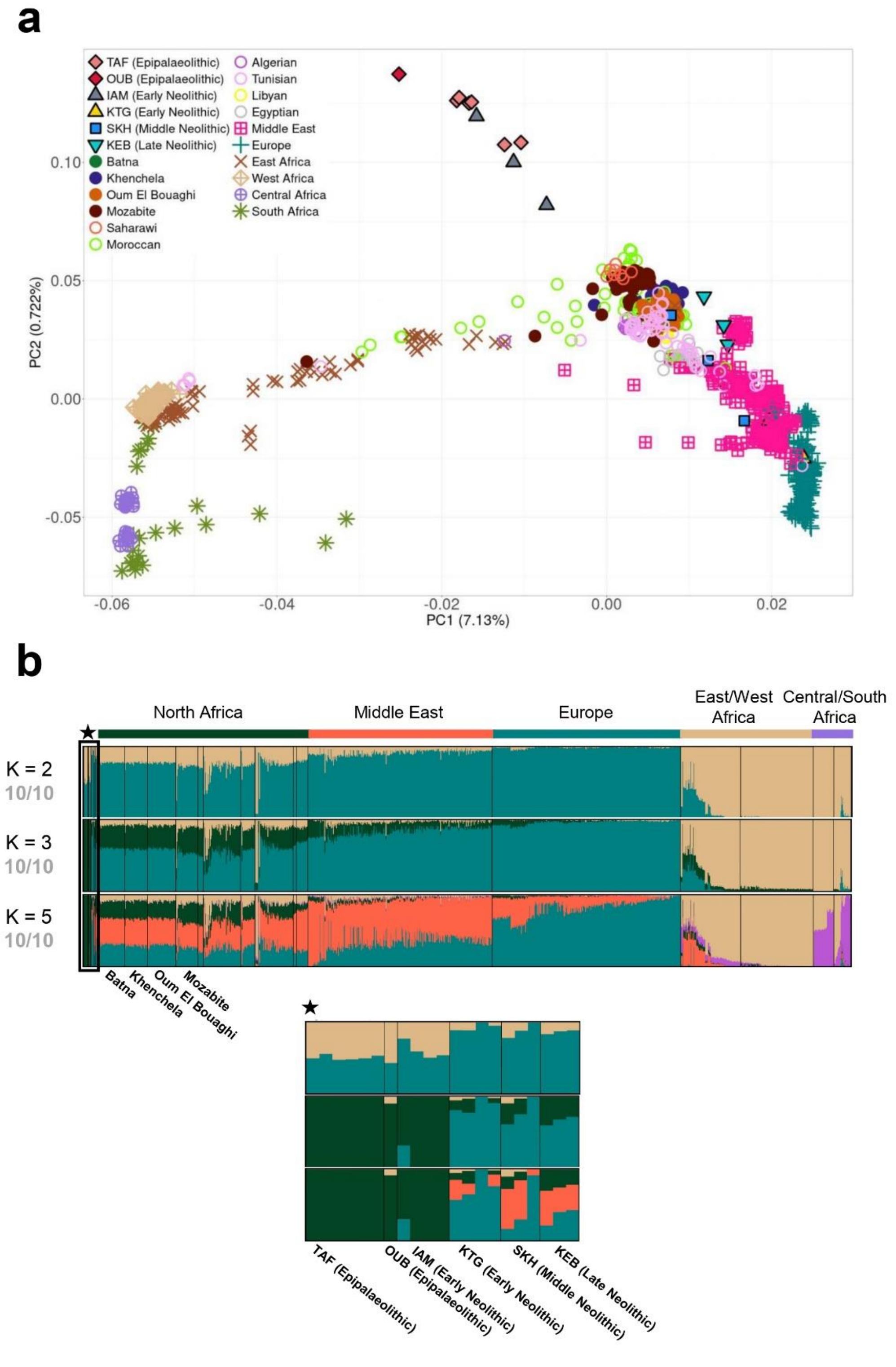

Supplementary Figure S3. PCA and ADMIXTURE results after including ancient samples in the *HO dataset* (see Methods). (a) PCA results, plotting PC1 against PC2, with Amazigh individuals represented by filled dots and ancient samples represented with a black contour and different shapes depending on their time periods. (b) ADMIXTURE analysis where each plot reflects each K's major mode. In gray, the number of runs out of 10 classified in the major mode at each value of K. K=5 has the lowest cross-validation error.

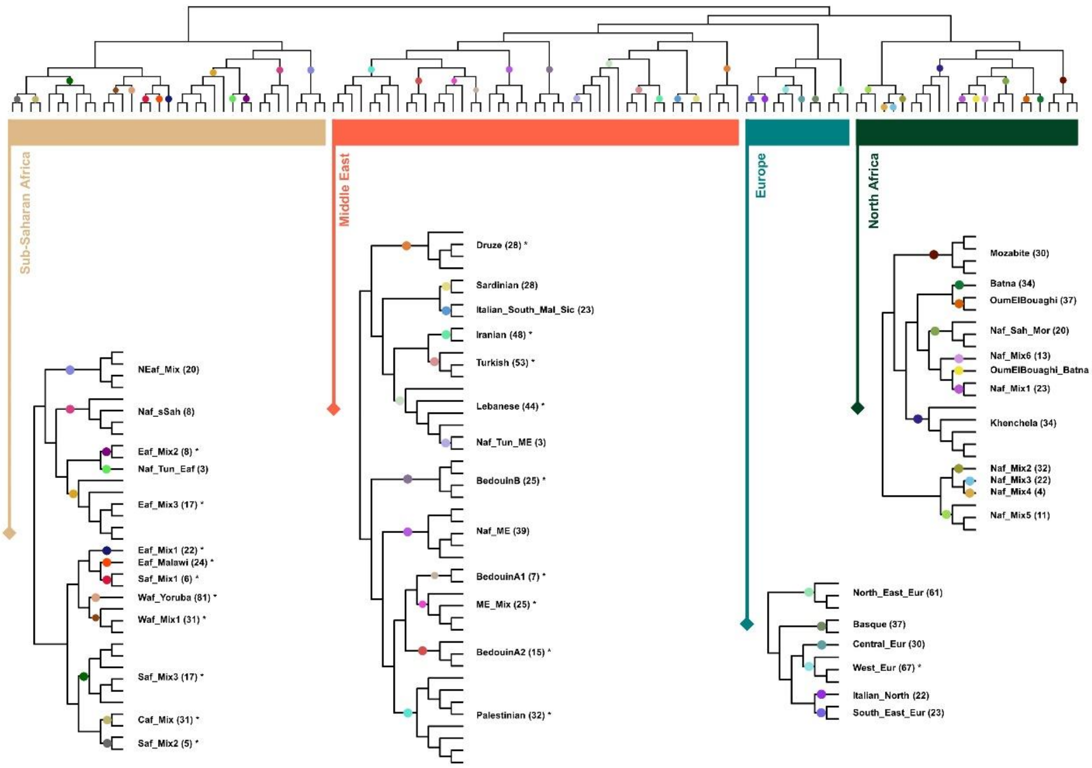

Supplementary Figure S4. Dendrogram obtained with fineSTRUCTURE from the chunkcounts coancestry matrix of ChromoPainter. It was used to classify the samples in genetic clusters, based on genetic similarity. This figure shows 4 general branches, and a closer look at these ones, shows the genetic clusters defined at the height of each dot, in total 44.

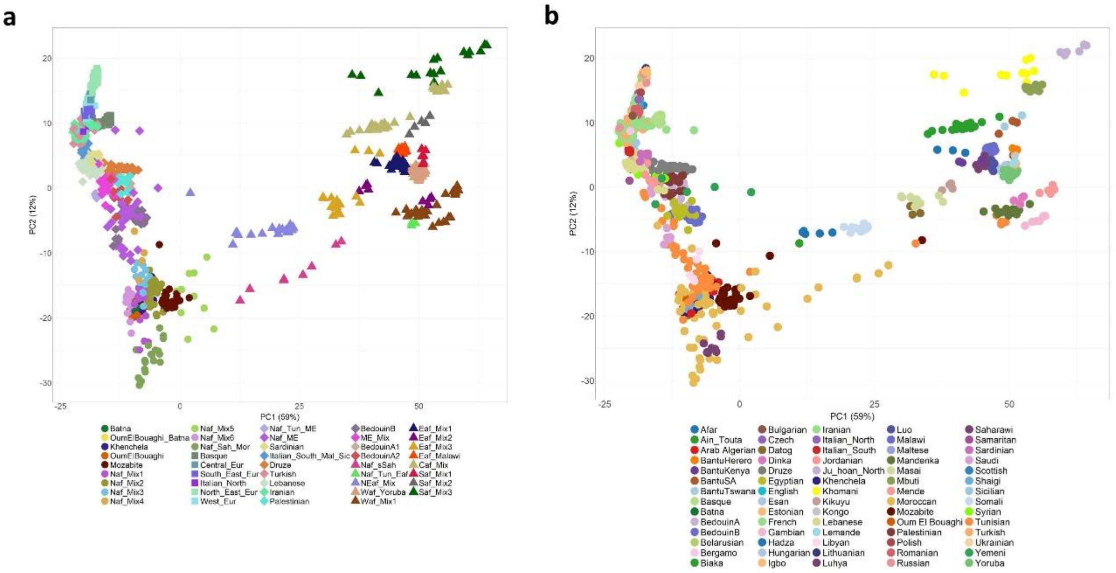

Supplementary Figure S5. PCAs computed for the chunkcounts output matrix from ChromoPainter, where in (**a**) the samples are classified in genetic clusters (colors) and general branches (●: North Africa, ■: Europe, ◆: Middle East and ▲: Sub-Saharan Africa) defined with fineSTRUCTURE, and in (**b**) they are classified with the population labels.

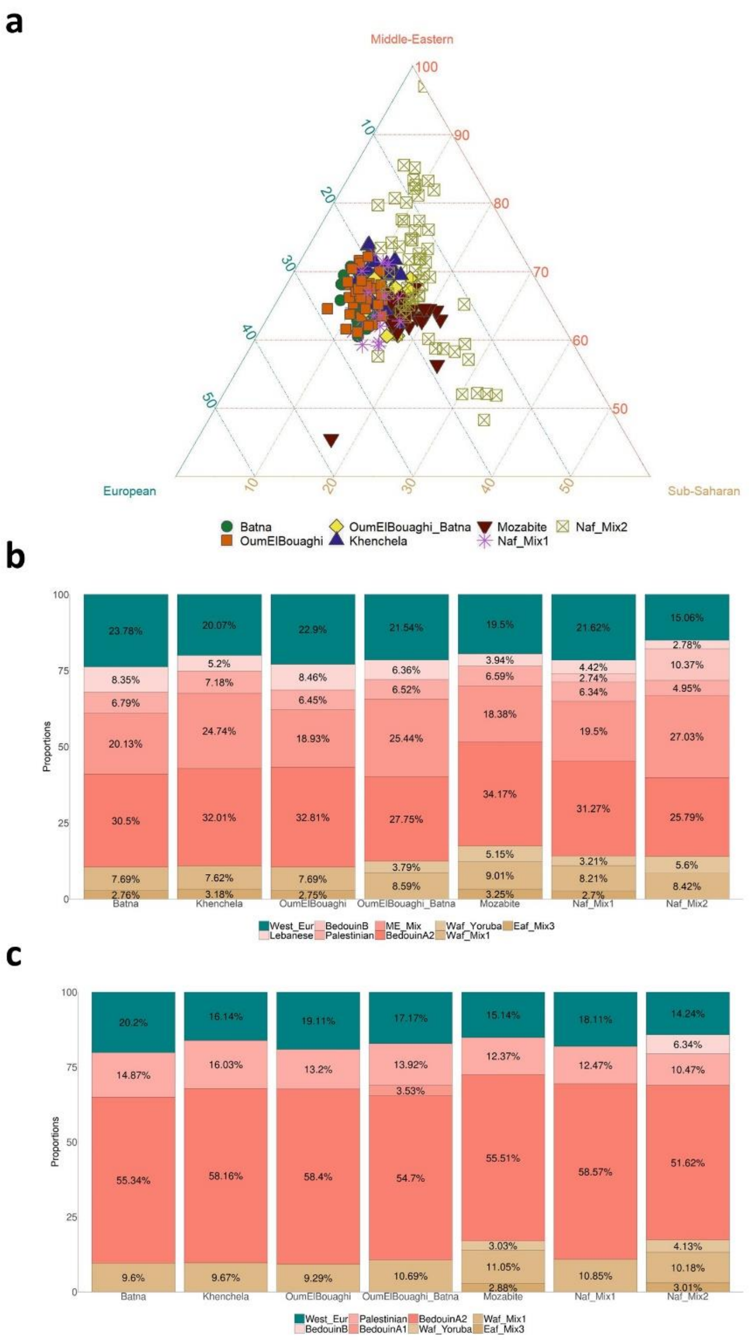

Supplementary Figure S6. Ancestry proportions obtained from the NNLS method and SOURCEFINDv2. (**a**) Ternary plot displaying the proportions obtained with the NNLS method for each individual considering three possible ancestries: Sub-Saharan, European, and Middle Eastern. (**b**) Barplot showing the average proportions obtained with the NNLS method of each donor group per target genetic cluster, considering proportions > 2.5%. (**c**) Barplot showing the average proportions obtained with SOURCEFINDv2 of each donor group per target genetic cluster, considering proportions > 2.5%.

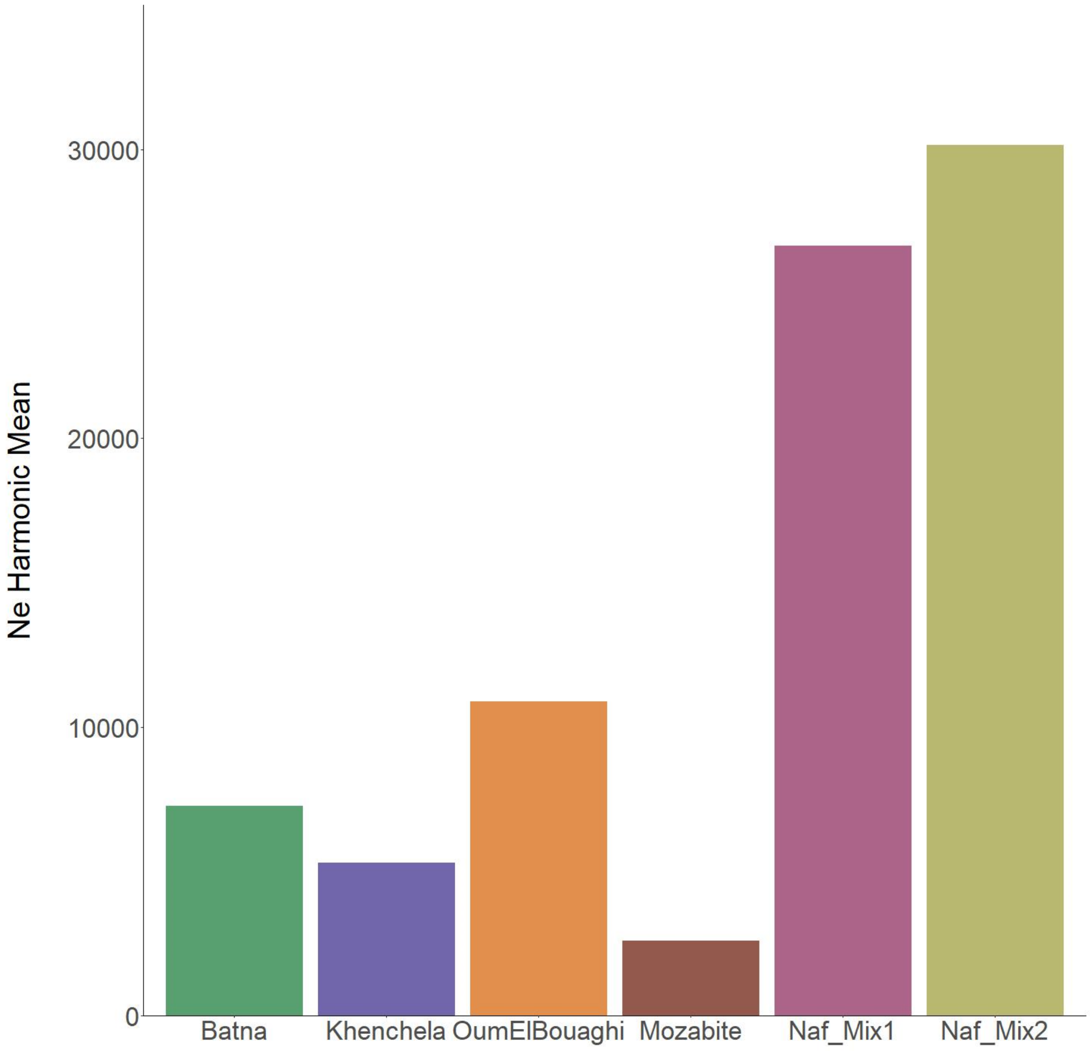

Supplementary Figure S7. Harmonic mean of the Effective Population Size (Ne). Harmonic mean computed for the Ne inferences over the last 100 generations.

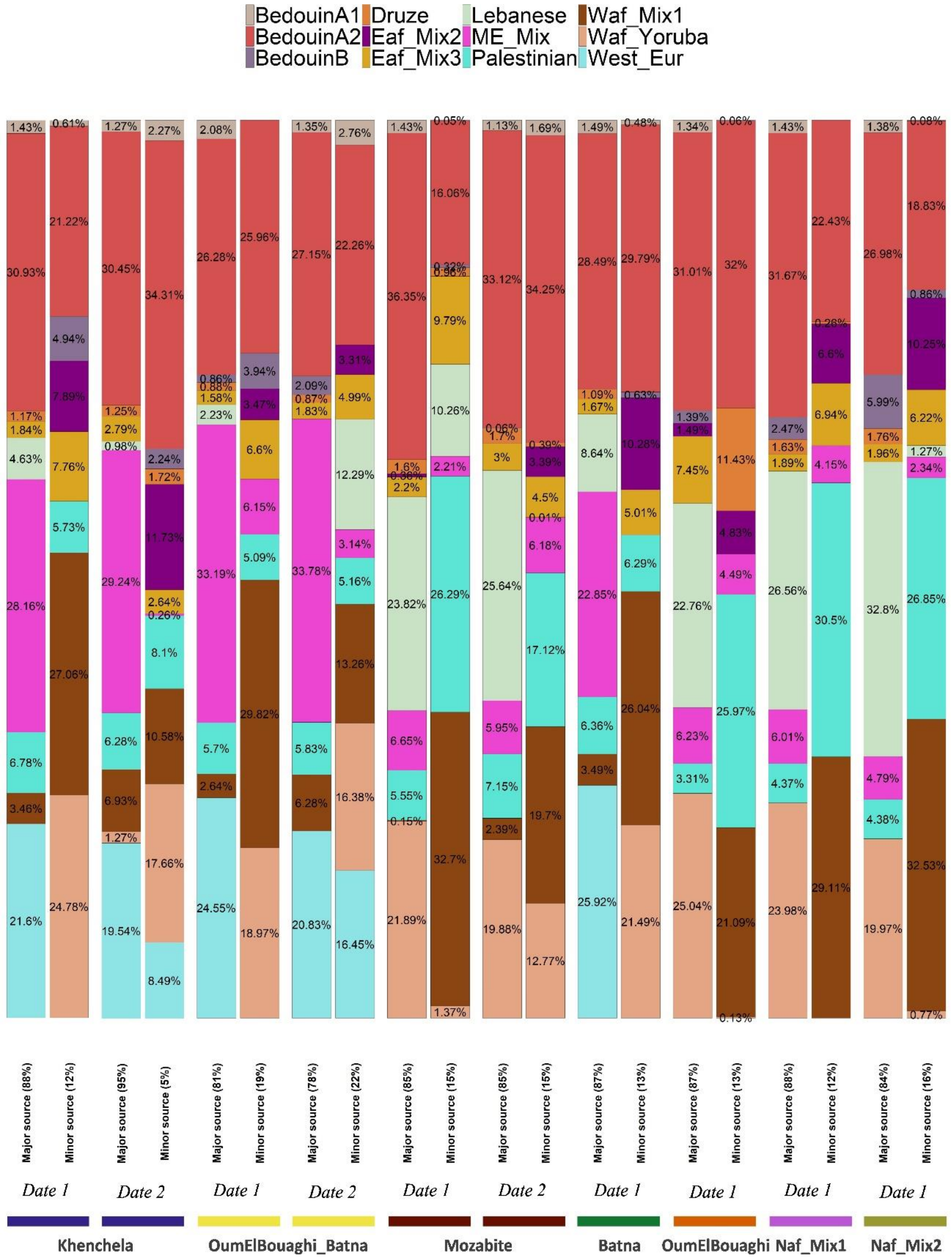

Supplementary Figure S8. Source populations inferred with GLOBETROTTER for each admixture event and target population, including the percentages of each surrogate population and the contribution of the sources.

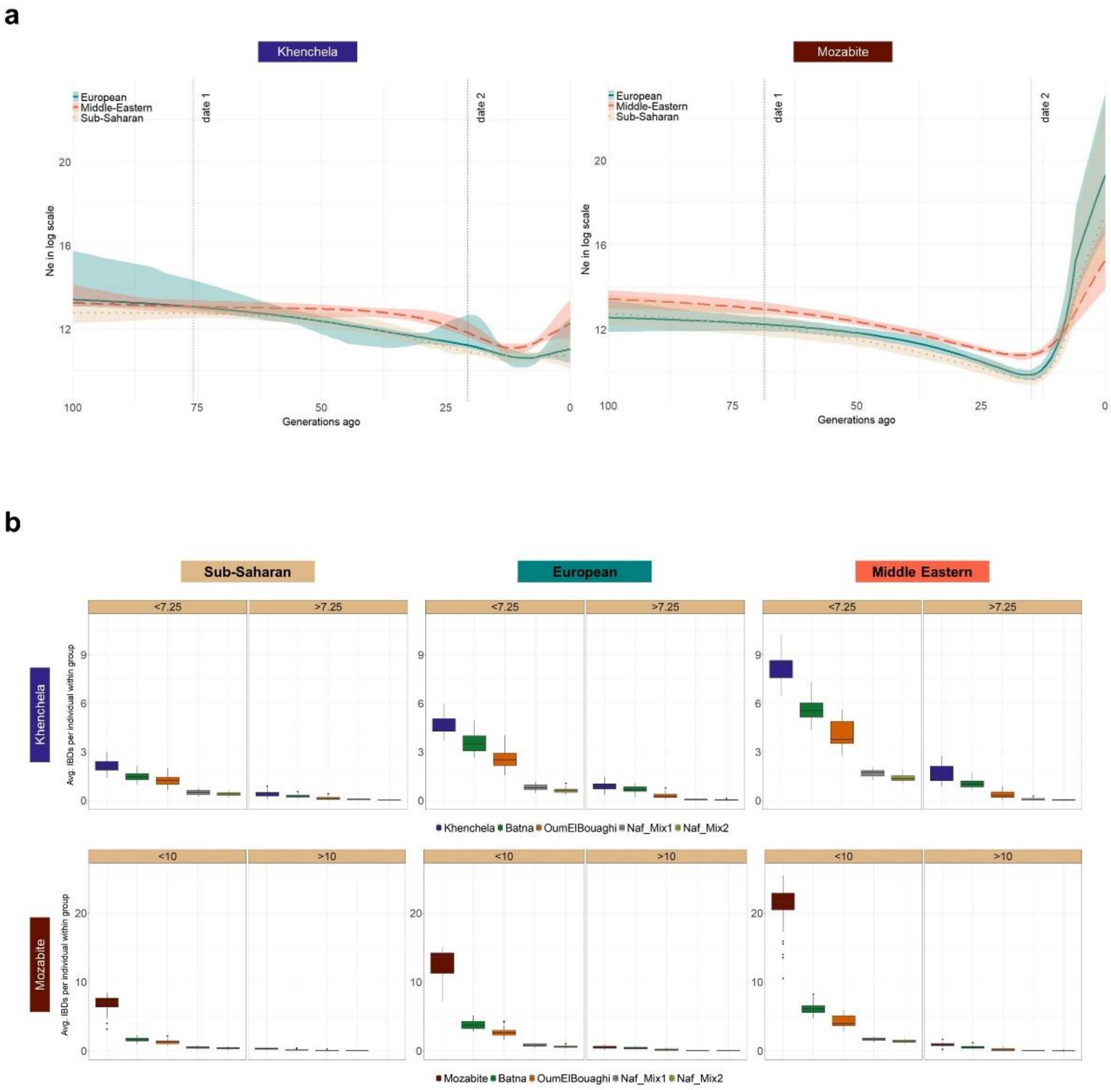

Supplementary Figure S9. Ancestry-specific inference of IBD segments. (**a**) Ancestry-specific Effective Population Size (Ne) over the last 100 generations, the dotted lines indicate the start of the admixture events detected for each specific cluster. (**b**) asIBDs before and after the most recent admixture event, for the *Khenchela* and *Mozabite* clusters, seen as IBDs shorter or longer than 7.25 and 10 cM, respectively, compared to other North African clusters for which only one admixture event was detected.
